# Supplementary material for: Prostaglandin E2 Exposure Disrupts E-Cadherin/Caveolin-1-Mediated Tumor Suppression to Favor Caveolin-1-Enhanced Migration, Invasion, and Metastasis in Melanoma Models
Source: Int J Mol Sci. 2023 Nov 29;24(23):16947. doi: 10.3390/ijms242316947 (PMC10707163; doi:10.3390/ijms242316947)
Supplement: Supplementary file 1 [file ijms-24-16947-s001.zip › ijms-2626940-supplementary.pdf]

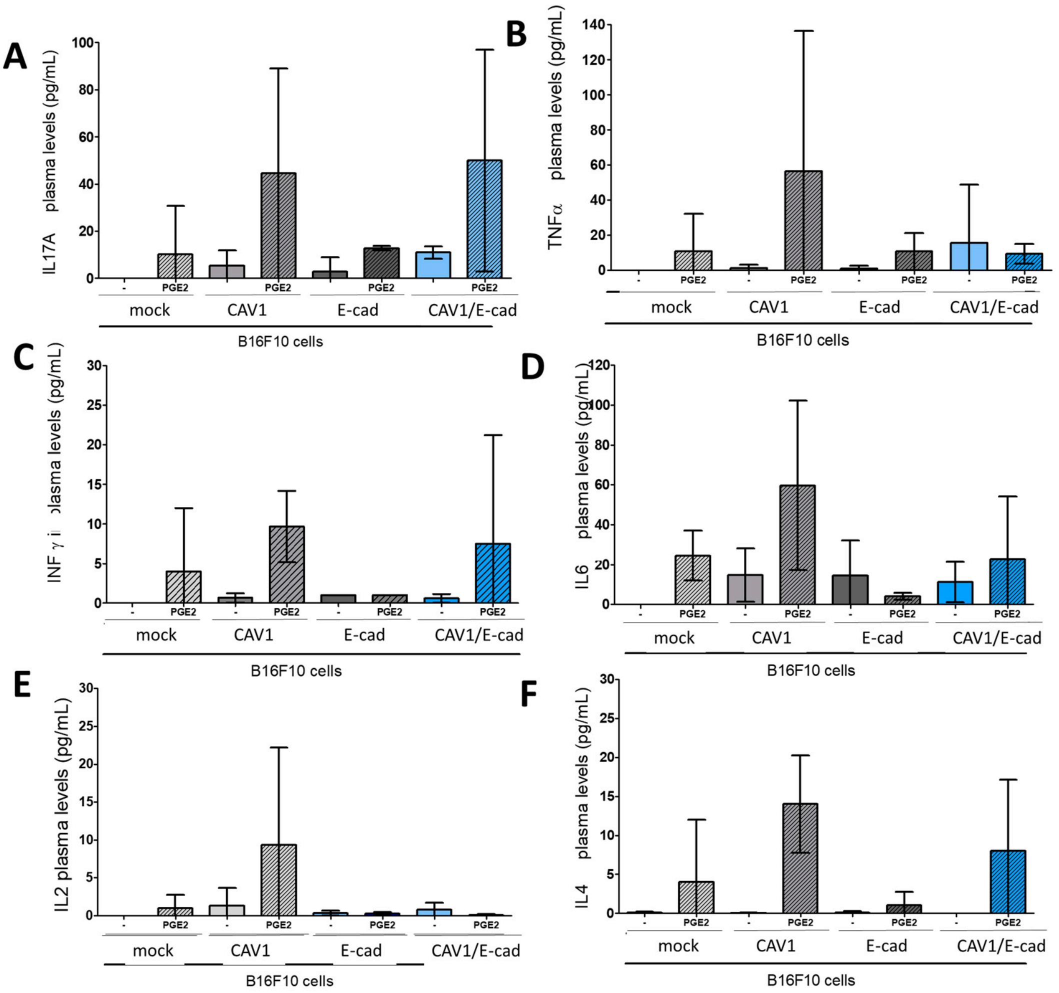

Figure S1: The cytokine presents in the plasma not shown inflammatory behavior in mice models.

When animals were euthanized, blood was collected from each mouse and the serum levels of the pro-inflammatory cytokines A) IL-10, B) TNFα, C) IFNγ, D) IL-6, E) IL-12 and F) IL4 were measured by cytometry. The assays were performed using the Mouse Inflammatory kit®, BD, (Catalog 552364). In addition, in these assays, body weight was not affected (data not shown). Thus, the differences observed for tumor formation and metastasis were not attributable to systemic changes in the levels of pro-inflammatory cytokines. The serum levels of interleukin-6 (IL-6), interleukin-10 (IL-10), interleukin-12 (IL-12), interferon-γ (IFN-γ), tumor necrosis factor (TNF), and interleukin-4 (IL-4) of mice that were previously injected subcutaneously with B16F10 (Mock), B16F10 (CAV1), B16F10 (E-cad), and B16F10 (CAV1/E-cad) cells, pre-stimulated or not with 50 μM PGE2 for 16 h, were determined by sandwich ELISA (BD Biosciences, San Diego, CA), according to the manufacturer's instructions.

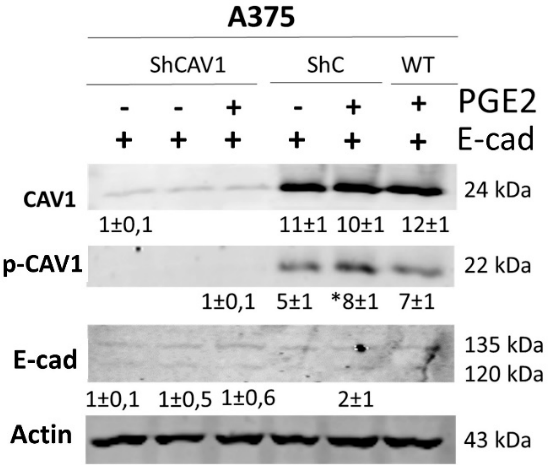

Figure S2: PGE2 increases the levels of phospho-CAV1 (pY14-CAV1).

Protein extracts from A375 (Mock), A375 (CAV1), A375 (E-cad), and A375 (E-cad/CAV1) cells treated or not with 20 µM PGE2 or vehicle (control) for 24 h. Phospho-CAV1 (pY14-CAV1) and CAV1 levels were evaluated by Western blotting. Results from a representative experiment (n = 3; ; *p < 0.05) are shown.
